# Fast and accurate average genome size and 16S rRNA gene average copy number computation in metagenomic data

**DOI:** 10.1186/s12859-019-3031-y

**Published:** 2019-09-05

**Authors:** Emiliano Pereira-Flores, Frank Oliver Glöckner, Antonio Fernandez-Guerra

**Affiliations:** 1 Microbial Genomics and Bioinformatics Research Group, Max Planck Institute for Marine Microbiology, Celsiusstraße 1, 28359 Bremen, Germany; 2 Department of Life Sciences and Chemistry, Jacobs University Bremen gGmbH, Campus Ring 1, 28759 Bremen, Germany; 3 Alfred Wegener Institute - Helmholtz Center for Polar- and Marine Research, Am Handelshafen 12, 27570 Bremerhaven, Germany; 4 Oxford e-Research Centre, University of Oxford, Oxford, OX1 3QG UK

**Keywords:** Microbial ecology, Metagenomics, Functional traits, Average genome size, 16S rRNA gene average copy number

## Abstract

**Background:**

Metagenomics caused a quantum leap in microbial ecology. However, the inherent size and complexity of metagenomic data limit its interpretation. The quantification of metagenomic traits in metagenomic analysis workflows has the potential to improve the exploitation of metagenomic data. Metagenomic traits are organisms’ characteristics linked to their performance. They are measured at the genomic level taking a random sample of individuals in a community. As such, these traits provide valuable information to uncover microorganisms’ ecological patterns. The Average Genome Size (AGS) and the 16S rRNA gene Average Copy Number (ACN) are two highly informative metagenomic traits that reflect microorganisms’ ecological strategies as well as the environmental conditions they inhabit.

**Results:**

Here, we present the ags.sh and acn.sh tools, which analytically derive the AGS and ACN metagenomic traits. These tools represent an advance on previous approaches to compute the AGS and ACN traits. Benchmarking shows that ags.sh is up to 11 times faster than state-of-the-art tools dedicated to the estimation AGS. Both ags.sh and acn.sh show comparable or higher accuracy than existing tools used to estimate these traits. To exemplify the applicability of both tools, we analyzed the 139 prokaryotic metagenomes of TARA Oceans and revealed the ecological strategies associated with different water layers.

**Conclusion:**

We took advantage of recent advances in gene annotation to develop the ags.sh and acn.sh tools to combine easy tool usage with fast and accurate performance. Our tools compute the AGS and ACN metagenomic traits on unassembled metagenomes and allow researchers to improve their metagenomic data analysis to gain deeper insights into microorganisms’ ecology. The ags.sh and acn.sh tools are publicly available using Docker container technology at https://github.com/pereiramemo/AGS-and-ACN-tools.

**Electronic supplementary material:**

The online version of this article (10.1186/s12859-019-3031-y) contains supplementary material, which is available to authorized users.

## Background

Advances in high-throughput sequencing technologies have pushed forward metagenomic studies, allowing the generation of massive amounts of data. As a consequence, metagenomics has become crucial to study microorganisms’ ecology [1]. Nonetheless, making sense of the metagenomic data is a complex and computationally intensive task. Commonly, metagenomes consist of many short-read sequences obtained from numerous different species, many of which are unknown.

Functional trait based-analyses offer an opportunity to improve our understanding of microorganisms’ ecology [2–4]. In particular, community functional traits measured at the genome level in a random sample of individuals (i.e., metagenomic traits), can help to uncover ecological patterns in short-read metagenomic data [5]. Functional traits are defined as characteristics of an organism that are linked to its performance, and consequently, influence its ecology and evolution [6]. Previous studies have used metagenomic traits to explain different aspects of microbial ecology, including why microorganisms live in a particular environment or how they respond to environmental changes [7–9].

The Average Genome Size (AGS) and the 16S rRNA gene Average Copy Number (ACN) are two metagenomic traits that can be computed from unassembled metagenomic data and provide valuable information to study the ecology of microbes. The genome size is known to be associated with environmental complexity and the organisms’ lifestyle [9–11]. Larger genomes tend to contain a more diverse metabolic repertoire, which in turn allows organisms to metabolize a greater diversity of substrates and inhabit heterogeneous environments [12]. Further, the AGS of a metagenome is important from a statistical perspective: the larger the AGS, the lower is the probability of sampling a specific gene. Hence, in order to avoid potential biases, this trait should be taken into account in gene-centric comparative metagenomics [13]. Lastly, the AGS can be used to estimate the proportion of an average-sized genome that has been sequenced to exhaustion, which can help to determine an appropriate sequencing depth, in particular when the aim is to generate metagenome-assembled genomes (MAGs) [14]. On the other hand, the 16S rRNA gene average copy number provides additional insights into the ecology of microorganisms. The 16S rRNA gene copy number in prokaryote genomes is known to vary from 1 to 15 [15]. This trait is associated with different growth strategies: organisms with low copy numbers tend to utilize resources more efficiently and inhabit oligotrophic environments, while those with high copy numbers can grow more rapidly under favorable conditions [16–18].

Currently, there are two publicly available tools dedicated to the computation of the AGS in metagenomes: the Genome relative Abundance and Average Size (GAAS) [19] and MicrobeCensus [20]. GAAS computes the AGS based on a BLAST search [21] against a reference database of microbial genomes. It was the first tool developed for the computation of the AGS, and although useful at its time, the runtime renders it highly impractical due to the now available large volume of metagenomes. Also, the fact that GAAS relies on genome databases to estimate the AGS, limits its accuracy when analyzing metagenomic samples containing novel taxa [20]. Alternatively, MicrobeCensus computes the AGS based on the abundance information of 30 universally distributed single-copy genes, following an approach initially proposed by Raes [22]: the AGS is estimated based on the abundance-weighted average of these marker genes, using optimized gene weights and empirically determined proportionality constants. Although MicrobeCensus has been shown to be considerably more accurate and faster than GAAS, the rapid increase of data generated by high-throughput sequencing technologies can still challenge its applicability.

For the ACN estimation, there are three publicly available tools able to predict this trait in metagenomic and amplicon data (i.e., PICRUSt [23], CopyRighter [24] and PAPRICA [25]). The approaches implemented in these tools are based on the work of Kembel et al. (2012) [26], which showed that the 16S rRNA gene copy number can be predicted based on the phylogenetic relationships of environmental sequences to reference organisms with known gene copy numbers. Although these tools can be used to estimate the ACN, their objective is to correct for copy number counts when estimating organisms’ abundances. They comprise a series of computationally intensive tasks, and their accuracy has been shown to be limited when analyzing taxa for which there are no close representatives in the reference phylogenies [27].

In this work, we developed two tools, which analytically derive the Average Genome Size (AGS) and 16S rRNA gene Average Copy Number (ACN) in prokaryotic metagenomes (ags.sh and acn.sh, respectively). Our implementations exploit recent advances in gene annotation algorithms to make methodological improvements for the estimation of these traits. We show that the ags.sh and acn.sh tools can rapidly and accurately predict the AGS and ACN, respectively. Compared to other tools used to estimate these traits, ags.sh and acn.sh represent an improvement in terms of accuracy and computational speed. Lastly, we analyzed the AGS and ACN in the TARA Oceans dataset [28], where we demonstrate the applicability of our tools and the value of these traits to reveal the ecological strategies adopted by microbial communities to cope with different environmental conditions.

## Materials and methods

### Implementation

The ags.sh and acn.sh tools were written in AWK, Bash, and R, and are provided as command line applications.

#### Average genome size computation tool (ags.sh)

ags.sh computes the Average Genome Size (AGS) and Number of Genomes (NGs) in metagenomic samples, based on the annotation of 35 single-copy genes that are universally present in prokaryotes [22] (Additional file 1). The workflow of ags.sh consists of the following steps: 1) Short-read sequences are filtered by length and trimmed with BBDuk [29] (optional step); 2) Open Reading Frames (ORFs) are predicted in the short-read sequences with FragGeneScan-Plus [30, 31] (optional step); 3) Single-copy genes are annotated with UProC [32]; 4) The gene coverage is estimated as the total number of annotated base pairs divided by the gene length; 5) The NGs is computed as the mean coverage of the 35 single-copy genes (see Eq. 1); 6) The AGS is computed as the ratio of the total number of base pairs to the NGs (see Eq. 2).

1 $$ \mathrm{NGs}=\frac{1}{35}\sum \limits_{\mathrm{i}=1}^{35}\frac{\mathrm{gene}\_{\mathrm{bp}}_i}{\mathrm{gene}\_{\mathrm{length}}_{\mathrm{i}}} $$

2 $$ \mathrm{AGS}=\frac{\mathrm{total}\_\mathrm{bp}}{\mathrm{NGs}} $$

Where “gene_bp_i_” and “gene_length_i_” are the number of annotated base pairs and length of marker gene “i”, and “total_bp” is the total number of base pairs in the target metagenome.

To annotate the single-copy genes, we created an UProC database. We downloaded the eggNOG database version 4.5 [33], selected the amino acid sequences (full-alignment files) used to create the Hidden Markov Model profiles of the 35 single-copy genes, applied the SEG low complexity filtering tool of the NCBI Blast+ 2.2 Suite [21] on these sequences, and created the UProC database with the *uproc-makedb* command.

#### 16S rRNA gene average copy number computation tool (acn.sh)

The workflow implemented in the acn.sh tool, consists of annotating the 16S rRNA genes with SortMeRNA 2.0 [34], estimating the 16S rRNA gene coverage as the number of annotated base pairs divided by the 16S rRNA gene length, and computing the ACN as the ratio of the 16S rRNA gene coverage to the NGs (see Eq. 3). The 16S rRNA gene length in this tool is set to 1542 bp, which corresponds to the full-length 16S rRNA gene of *Escherichia coli*. To run SortMeRNA, we use its prepackaged silva-bac-16 s-id90 and silva-arc-16 s-id95 16S rRNA gene sequence databases.

3 $$ \mathrm{ACN}=\frac{16\mathrm{S}\_\mathrm{gene}\_\mathrm{bp}/16\mathrm{S}\_\mathrm{gene}\_\mathrm{length}}{\mathrm{NGs}} $$

Where “16S_gene_bp” and “16S_gene_length” are the number of annotated base pairs and the 16S rRNA gene length, respectively, and “NGs” is the number of genomes in the target metagenome.

### Data acquisition, pre-processing, and analysis

The 139 prokaryotic metagenomes of the TARA Oceans dataset were downloaded from the European Nucleotide Archive [35] (ENA:PRJEB1787). To pre-process the metagenomic short-read data, we applied the following procedure. We clipped the adapter sequences (obtained from Shinichi Sunagawa personal communication, July 21, 2015) with the BBDuk tool of the BBMap 35.00 suite [29]; We then merged the paired-end reads with VSEARCH 2.3.4 [36], quality trimmed all reads at Q20 and filtered out sequences shorter than 45 bp using BBDuk; Lastly, we de-replicated the quality-controlled sequences with VSEARCH.

We estimated the Average Genome Size (AGS) and the 16S rRNA gene Average Copy Number (ACN) in the 139 metagenomes with the ags.sh and acn.sh tools, respectively. To run the ags.sh tool we used the minimum length parameter set to 100 bp. To filter significant 16S rRNA gene sequence alignments when running the acn.sh tool, we used an e-value of 1e-5. We selected a matching subset of 63 TARA Oceans metagenomes representing the Surface (SRF), Deep Chlorophyll Maximum (DCM) and Mesopelagic (MES) water layers in 21 sampling stations [28], to analyze the changes of the AGS and ACN between water layers.

To test whether the AGS and ACN values differ between water layers, we applied a paired Wilcoxon rank-sum test between each pair of water layers, using the *wilcox.test* function of the vegan R package [37].

We used TARA Oceans’ taxonomic abundance profile computed by Sunagawa et al. 2015 based on the annotation of 16S rDNA Operational Taxonomic Units (OTUs), to search for genera that correlated with the AGS and ACN. First, we removed singletons and genera with a total relative abundance lower than 0.001%, and computed the genera relative abundance in each metagenome (total sum scaling standardization). Subsequently, we selected the genera for which their correlation with either of these traits had a *p*-value lower than 0.001 after applying the Bonferroni correction for multiple comparisons. Additionally, we compared the AGS with the functional richness computed by Sunagawa et al. in the 139 TARA Oceans metagenomes.

### Simulation of metagenomic datasets

To assess the performance of our tools, we created three simulated metagenomic datasets (i.e., General, Infant Gut, and Marine). Each dataset is composed of 50 metagenomes, and all metagenomes have a size of two million reads. The metagenomes of the General dataset were simulated based on a random selection of prokaryotic species. The Infant Gut and Marine datasets approximate the taxonomic composition of the microbial communities found in the infant’s gut and marine environments, respectively.

To create each of the three simulated metagenomic datasets, we first created a reference dataset of complete genome sequences and the abundance profiles to define the community composition of each metagenome. In the case of the General genome reference dataset, we randomly selected 500 genera from all the prokaryotic genera in the NCBI RefSeq database [38] and downloaded a maximum of three genome sequences per genus with an assembly status of “Complete genome” (on November 8, 2017). The resulting dataset comprised 751 different species. For the Infant Gut genome reference dataset, we used the genus taxonomic annotation of the metagenome-assembled genomes (MAGs) generated by Sharon et al. (2013) [39] obtained from fecal samples collected from a premature infant, to guide the selection of species. We downloaded for each species one genome with an assembly status of “Complete genome” from the NCBI RefSeq database. If a species did not have a complete representative genome, we randomly selected another species with the same genus affiliation. The Infant Gut reference genome dataset contains 95 different species. Finally, the Marine reference genome dataset was created based on the taxonomic composition of TARA Oceans 16S rDNA Operational Taxonomic Units (OTUs) described by [28]. We selected 172 genera for which there was at least one representative completely sequenced genome and downloaded a maximum of three genomes per genus from RefSeq, irrespective of their species affiliation. This database comprises 308 species.

To define the community profile of each metagenome, we randomly selected between 20 and 80 genomes from a reference dataset and assigned the genome abundances by sampling from a lognormal distribution with mean 1 and standard deviation of 0.5. We used these profiles together with the corresponding reference genome sequence data as an input to run MetaSim v0.9.5 [40], where we set the read length to 300 bp and the substitution rate to 1 × 10^− 3^.

Lastly, we simulated a second Marine metagenomic dataset of 50 metagenomes (i.e., Marine dataset-2). This dataset was generated using the marine abundance profiles and reference genome dataset mentioned above; however, in this case, we simulated merged paired-end reads and varied their length according to the following distribution: p(50 bp) = 0.05; p(110 bp) = 0.15; p(150 bp) = 0.15; p(165 bp) = 0.5; p(180 bp) = 0.15. In addition, the substitution rate along each read was set to increase from 1 × 10^− 4^ to 9.9 × 10^− 2^. The simulated short-read sequences were merged using VSEARCH [36] with default parameters. With this read length distribution and error rates, we aimed to generate a more realistic dataset. It has similar characteristics as the metagenomic data obtained using Illumina HiSeq 2000 sequencing technology (as the prokaryotic metagenomes of the TARA Oceans dataset), and the majority of Illumina sequencing technologies in general [41].

In Additional file 2, we show the taxonomic composition of the reference datasets of complete genome sequences, and in Additional file 3, we show details of the simulated metagenomic datasets.

### Benchmarking and validation

To benchmark the wall-clock runtime of ags.sh against MicrobeCensus, we randomly selected five (pre-processed) metagenomes of the TARA Oceans dataset and subsampled these to two million paired-end reads with the seqtk v1 tool [42]. Next, we ran the AGS computation on each metagenome three times with both tools, using a different number of threads in each run (i.e., 4, 8, and 16). All the computations were performed in a workstation with Intel(R) Xeon(R) CPU E7–4820 v4 @ 2.00GHz.

To benchmark the accuracy of ags.sh against MicrobeCensus, we computed the AGS in the metagenomes of the General, Infant Gut, and Marine simulated datasets with both tools and compared it with the real AGS. To assess the accuracy of these tools in relation to the read length, we trimmed the 3′ end of the reads to simulate metagenomic datasets of different read lengths. Namely, for each of the General, Marine, and Infant Gut datasets, we trimmed the simulated 300 bp reads to 100 bp, 120 bp, 130 bp, 140 bp, 150 bp, 160 bp, 170 bp, 180 bp, and 200 bp. We processed a total of 500 metagenomes per dataset.

In these analyses, ags.sh was run with default parameters and MicrobeCensus was set to process the total number of reads in a metagenome. To derive the real AGS of a simulated metagenome, we computed the sum of the lengths of its component genomes weighted by their respective abundance, divided by the total abundance of genomes (see Eq. 4). The genome abundances were obtained from the abundance profiles used to simulate the metagenomes. To quantify the accuracy, we computed the Pearson’s correlation and Absolute Percentage Error (APE) (see Eq. 5) of the AGS computed by ags.sh and MicrobeCensus, with respect to the real AGS.

4 $$ {\mathrm{AGS}}_{\mathrm{real}}=\frac{1}{\mathrm{total}\_\mathrm{abund}}\sum \limits_{\mathrm{i}=1}^{\mathrm{k}}\mathrm{genome}\_{\mathrm{length}}_{\mathrm{i}}\times \mathrm{genome}\_{\mathrm{abund}}_{\mathrm{i}} $$

Where “total_abund” is the total abundance in the target metagenome, and “genome_length_i_” and “genome_abund_i_” are the length and abundance of genome “i”, respectively.

5 $$ \mathrm{APE}=100\times \left|\frac{\mathrm{Ref}-\mathrm{Est}}{\mathrm{Ref}}\right| $$

Where “Ref” and “Est” are the reference and estimated trait values, respectively.

To compare ags.sh vs. MicrobeCensus on real data, we computed the AGS with these tools on a randomly selected subset of 50 (pre-processed) metagenomes of TARA Oceans. To accelerate the computation of the AGS, we randomly subsampled the metagenomes to two million reads using the seqtk v1 tool [42].

To measure the wall-clock running time of acn.sh, we used the same five TARA Oceans metagenomes subsampled to two million paired-end reads and previously used to benchmark the ags.sh running time. Next, we ran acn.sh using different thread numbers (i.e., 4, 8, and 16) and measured the wall-clock running time. These computations were performed in a workstation with Intel(R) Xeon(R) CPU E7–4820 v4 @ 2.00 GHz.

To benchmark the accuracy of acn.sh, we compared it with PICRUSt [23], CopyRighter [24], and PAPRICA [25]. We computed the ACN with these four tools in the three simulated metagenomic datasets, where we also trimmed the 3′ end of the metagenomic reads to produce different read lengths (i.e., 100 bp, 150 bp, 200 bp, and 300 bp). We then computed the Pearson’s correlation and APE between the predicted and real ACNs.

To compute the ACN with acn.sh, we ran the tool with default parameters. To compute the ACN with CopyRighter and PICRUSt, we applied the following procedure: 1) Reads containing 16S rRNA genes were identified with SortMeRNA 2.0 [34]; 2) 16S rRNA gene sequences were extracted and clustered at 99% identity with VSEARCH 2.3.4 [36]; 3) Cluster centroid sequences were blasted against the GreenGenes databases GG2012 (release October 2012) and GG2013 (release May 2013) [43], using BLASTN [21] with an e-value of 0.001 and an identity threshold of 99%; 4) The 16S rRNA gene copy numbers of the best hits were parsed out of the respective lookup tables (ssu_img40_gg201210.txt and 16S_13_5_precalculated.tab for CopyRighter and PICRUSt, downloaded from [44, 45], respectively); 5) The ACN was computed as the average of the predicted 16S rRNA gene copy number (i.e., 16S rRNA gene copy number of the best hits), weighted by the abundance of the cluster represented by the respective query sequence (see Eq. 6).

To compute the ACN with PAPRICA, we used the cluster centroid sequences to run the paprica-run.sh script using the –large parameter for the paprica-place_it.py script (i.e., to increase the memory utilization). Then, we computed the average of the 16S rRNA gene copy numbers predicted for these sequences, weighted by the respective cluster abundances (see Eq. 6).

6 $$ {\mathrm{ACN}}_{\mathrm{est}}=\frac{1}{\mathrm{total}\_\mathrm{abund}}\sum \limits_{\mathrm{i}=1}^{\mathrm{k}}\mathrm{pred}\_\mathrm{copy}\_{\mathrm{num}}_{\mathrm{i}}\times \mathrm{cluster}\_{\mathrm{abund}}_{\mathrm{i}} $$

Where “pred_copy_num_i_” and “cluster_abund_i_” are the predicted 16S rRNA gene copy number and cluster size of query sequence “i”, and “total_abund”, is the total number of identified 16S rRNA gene sequences in a metagenome.

Similarly, as described above, we computed the real ACN of a metagenome, as the sum of the 16S rRNA gene copy numbers of its component genomes weighted by their respective abundance and divided by the total abundance of genomes (see Eq. 7). The 16S rRNA gene copy numbers were obtained from the NCBI features annotation.

7 $$ {\mathrm{ACN}}_{\mathrm{real}}=\frac{1}{\mathrm{total}\_\mathrm{abund}}\sum \limits_{\mathrm{i}=1}^{\mathrm{k}}\mathrm{genome}\_\mathrm{copy}\_{\mathrm{num}}_{\mathrm{i}}\times \mathrm{g}\mathrm{enome}\_{\mathrm{abund}}_{\mathrm{i}} $$

Where the “total_abund” is the total abundance in the target metagenome, and “genome_copy_num_i_” and “genome_abund_i_” are the 16S rRNA gene copy number and abundance of genome “i”, respectively.

## Results and discussion

**Fig. 1:**
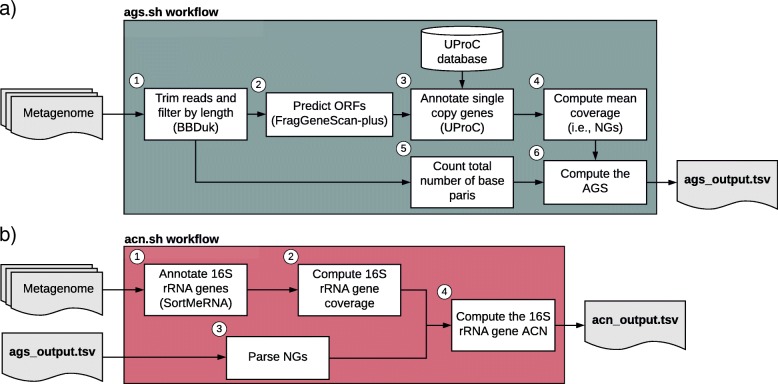
Workflows implemented in the ags.sh and acn.sh tools. **a** ags.sh workflow consists of the following steps: 1) Filtering out and trimming reads to obtain an appropriate read length range using the BBduk tool [29] (optional step); 2) Predicting the Open Reading Frames (ORFs) with FragGeneScan-plus [30] (optional step); 3) Annotating the single-copy genes in the ORF’s amino acid sequences with UProC [32]; 4) Computing the Number of Genomes (NGs) as the mean gene coverage of the single-copy genes; 5) Counting the total number of base pairs; 6) Computing the Average Genome Size (AGS) as the ratio of the total number of base pairs to the NGs. **b** The tasks performed by acn.sh are as follows: 1) Annotating the 16S rRNA genes with SortMeRNA [34]; 2) Computing the 16S rRNA gene coverage as the number of annotated base pairs divided by the 16S rRNA gene length; 3) Parsing the NGs from the ags.sh output; 4) Computing the ratio of the 16S rRNA gene coverage to the NGs to derive the 16S rRNA gene Average Copy Number (ACN)

Our implementation to compute the AGS is based on the annotation of 35 prokaryotic universally distributed single-copy genes identified by Raes et al., (2007) [22]. Most of these genes are part of the translation machinery and essential for cellular life. The main finding that allowed us to develop ags.sh (and in turn acn.sh), is that the annotation of the 35 marker genes in unassembled metagenomes, using new, fast and accurate tools, can be used to rapidly estimate the genes’ coverage, which accurately approximates the total number of genomes (NGs). Thus, we can derive the AGS analytically, as the ratio of NGs and the total number of base pairs in a metagenome. However, to estimate the NGs it is crucial to perform a precise annotation of the single-copy genes. To this end, we include in ags.sh an option to filter and trim sequence reads to obtain the optimal read lengths for the annotation of single-copy genes (see Fig. 1a). The computation of the 16S rRNA gene average copy number follows a similar methodology: we estimate the coverage of the 16S rRNA genes and divided it by the NGs (see Fig. 1b).

### Benchmarking the average genome size computation tool (ags.sh)

**Fig. 2:**
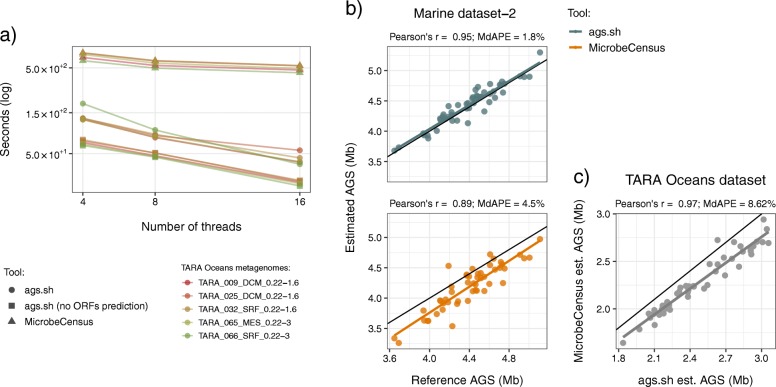
Benchmarking the running time and accuracy ags.sh against MicrobeCensus. **a** Plot comparing the running time of ags.sh with MicrobeCensus. We compared the wall-clock runtime between both tools using 4, 8, and 16 threads, in five TARA Oceans metagenomes subsampled to two million paired-end reads. We also compared the ags.sh runtime using previously predicted Open Reading Frames (ORFs). When the ORF prediction procedure was included, ags.sh was 11 times faster than MicrobeCensus using 16 threads. **b** Scatter plots comparing the accuracy of the AGS computed by ags.sh (upper panel) and MicrobeCensus (lower panel) with the reference AGS in the metagenomes of the Marine dataset-2. **c** Scatter plot comparing the AGS computed by ags.sh and MicrobeCensus in 50 TARA Oceans metagenomes, randomly subsampled to two million reads. The black line shown in the scatter plots from b) and c) represents the one-to-one relationship. The absolute percentage error was computed as 100 x |(AGS_ref_ - AGS_est_)/AGS_ref_|, where AGS_est_ and AGS_ref_ are the estimated and reference AGS, respectively. MdAPE acronym stands for Median Absolute Percentage Error

Firstly, we benchmarked the wall-clock runtime of ags.sh against MicrobeCensus (see Fig. 2a and Additional file 4). We used a subset of five TARA Oceans metagenomes subsampled to two million paired-end reads (see Materials and Methods), to measure the runtime of these tools with increasing number of threads (i.e., 4, 8 and 16). In this analysis, we also benchmarked the runtime of ags.sh using previously predicted Open Reading Frame (ORF) amino acid sequences. We consider this is a likely scenario when using our tool, given that the prediction of ORFs is a standard procedure in most metagenomic analysis workflows. With and without the ORF prediction step, ags.sh was on average 6.6 and 12.6 times faster than MicrobeCensus, respectively. Ags.sh also showed a greater runtime improvement when we increased the number of threads from 4 to 16. The acceleration achieved by our implementation is the result of using a fork of FragGeneScan-plus [30, 31] and the UProC program [32] for the ORF prediction and gene annotation, respectively. FragGeneScan-plus is several times faster than FragGeneScan [46], and has the same prediction accuracy, while UProC is up to three orders of magnitude faster and more sensitive than profile-based methods on unassembled short-read sequences. Reducing the runtime also has consequences for the AGS estimation accuracy, given that all the metagenomic data available can be readily used to compute this trait.

Secondly, we compared the accuracy of ags.sh against MicrobeCensus. We used both tools to estimate the AGS in the simulated metagenomes of three different datasets we generated (i.e., General, Infant Gut, and Marine datasets; see Material and Methods). Simulated data allowed us to calculate the real AGS in the metagenomes, which we used as a reference to evaluate the accuracy of these tools. Each simulated dataset is composed of 50 metagenomes of two million reads of 300 bp, which were trimmed to different read lengths, to evaluate the accuracy of the tools as a function of the read length.

The analysis showed that the performance of both tools changed very little between datasets, and ags.sh had comparable or higher accuracy than MicrobeCensus in metagenomes with a read length between 120 and 200 bp (see Additional file 5 and Additional file 6). The Pearson’s correlation values showed marginal differences between both tools and remained practically constant within each dataset when the read length was changed. However, the error values revealed that the accuracy of ags.sh varies with the read length, with a trend of having higher accuracy than MicrobeCensus in metagenomes with a read length between 120 and 200 bp. MicrobeCensus was less affected by the read length and outperformed ags.sh in metagenomes with read lengths of 100 and 300 bp.

The optimal read length range observed for our tool reflects the read length in which UProC has an optimal sensitivity and specificity. In essence, if the reads are too short there is a lower sensitivity, and consequently, the AGS is overestimated (NGs underestimated). Conversely, when the reads are too long, there is a lower specificity and the AGS is underestimated (NGs overestimated).

It is important to point out that ags.sh has an option to remove and trim metagenomic reads to obtain appropriate read lengths. This way, it can always process metagenomic reads where it has its highest accuracy.

In addition, to test these tools in a more realistic scenario, we generated a second simulated Marine metagenomic dataset (i.e., Marine dataset-2). Marine dataset-2 is composed of 50 metagenomes of two million merged paired-end reads, with a length that varies from 100 to 180 bp, and with an error rate increasing along each read from 1 × 10^− 4^ to 9.9 × 10^− 2^. With such data characteristics, we simulate the data generated by Illumina HiSeq 2000 sequencing technology, and in particular, the characteristics of TARA Oceans metagenomes, which we analyzed as an example application of our tools (see below). In Fig. 2b, we show the scatter plots comparing the estimated AGS using ags.sh and MicrobeCensus, vs. the reference AGS. In this example, MicrobeCensus produced more biased estimates: the tool consistently underestimated the AGS. The Pearson’s correlation and median of the Absolute Percentage Errors (MdAPEs) were 1.8% and 0.95 for ags.sh, and 4.5% and 0.89 for MicrobeCensus, respectively.

Lastly, we compared the AGS obtained with ags.sh and MicrobeCensus on real data. We used these tools to compute the AGS in 50 metagenomes of the TARA Oceans dataset (Fig. 2c). To reduce the computation time, we randomly subsampled the metagenomes to two million reads. Although the AGSs computed with both tools were highly correlated (Pearson’s r = 0.97), MicrobeCensus showed a similar pattern regarding the underestimation of the AGS as observed in the simulated data, which indicates that the predictions of our tool are closer to the true AGS of these samples. The bias observed in MicrobeCensus estimates might be explained by the fact that this tool is based on a series of empirically determined constants, resulting in a somewhat limited generalization.

### Benchmarking the 16S rRNA gene average copy number estimation tool (acn.sh)

**Fig. 3:**
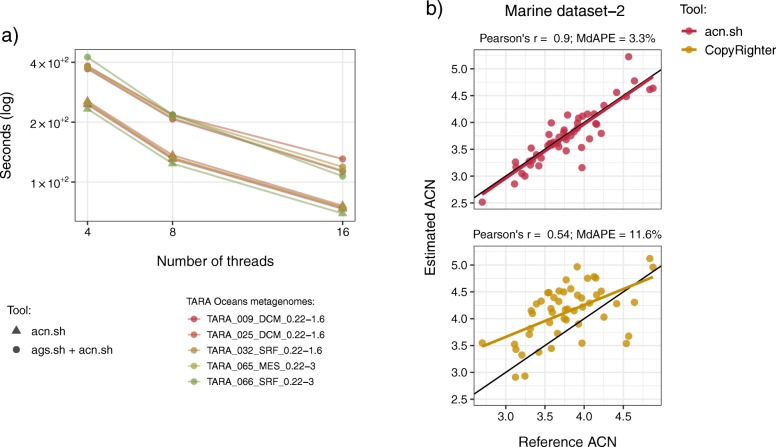
Evaluating the running time of acn.sh and benchmarking its accuracy against CopyRighter. **a** Plot showing the wall-clock runtime of acn.sh, and the running time of ags.sh plus acn.sh, using 4, 8, and 16 threads, for the computation of the 16S rRNA gene Average Copy Number (ACN) in five TARA Oceans metagenomes subsampled to two million paired-end reads. **b** Scatter plot comparing the ACN computed by acn.sh (upper panel) and CopyRighter (lower panel) with the reference ACN in the metagenomes of the Marine dataset-2. The black line shown in the plot represents the one-to-one relationship. Similarly as above, we applied the following formula to compute the absolute percentage error: 100 x |(ACN_ref_ - ACN_est_)/ACN_ref_|, where ACN_est_ and ACN_ref_ are the estimated and reference ACN, respectively

Analog to the previous benchmark analysis, we measured the wall-clock runtime of acn.sh using 4, 8, and 16 threads on the five subsampled metagenomes of TARA Oceans (see Fig. 3a and Additional file 7). In this evaluation, we observed that acn.sh scales very well with the number of threads and is able to process approximately one million reads per minute using eight threads. The most computationally intensive task performed by acn.sh, is the annotation of the 16S rRNA genes with SortMeRNA [34], which determines the runtime. SortMeRNA has an optimal accuracy-speed trade-off, making it very convenient for the computation of this trait in metagenomic data. In addition, given that acn.sh depends on the NGs estimated with ags.sh, we benchmarked the running time of ags.sh plus acn.sh. When both tools are taken into account, we observed that a two million paired-end reads metagenome is processed in less than four minutes using eight threads.

We also compared the accuracy of acn.sh with PICRUSt, CopyRighter, and PAPRICA (see Additional file 8 and Additional file 9). As described above, we used acn.sh to compute the ACN in the simulated metagenomes of the General, Infant Gut, and Marine datasets, trimmed to different read lengths. We then computed the real ACN, which we used as a reference to assess the accuracy of these tools. This analysis showed that acn.sh is considerably more accurate than the other three tools. As observed for ags.sh, acn.sh had comparable performance in the three datasets: while the correlation coefficients were not affected by the read length, the APE values increased in metagenomes of 100 and 300 bp. The under- and overestimation of the NGs computed by ags.sh limited the accuracy in these latter two cases. When considering an appropriate read length (i.e., 150 and 200 bp), the correlation and MdAPE values ranged from 0.9 to 0.94, and from 2.9 to 6.4%, respectively.

Conversely, the performance of PICRUSt, CopyRighter, and PAPRICA varied between datasets, especially between the Infant Gut and Marine datasets, where we observed their highest and lowest performance, respectively. For instance, the correlation and MdAPE values of PAPRICA, which showed the highest accuracy of the three in the Infant Gut dataset, went from 0.78 to 0.48, and from 3.2 to 15.8%, respectively, in the metagenomes of 100 bp. Such finding is likely to reflect the low representation of sequenced environmental taxa in reference phylogenies, which is known to limit the prediction power of these tools [27].

In Fig. 3b and c, we show the scatter plots comparing the ACN computed with acn.sh and CopyRighter vs. the reference ACN in the Marine dataset-2. We selected CopyRighter for this comparison since it performed (moderately) better than PICRUSt and PAPRICA in the Marine dataset. The scatter plots show the superiority of acn.sh for the computation of the ACN, particularly evidenced by the correlation coefficients (Pearson’s r = 0.9 and Pearson’s r = 0.54 for acn.sh and CopyRighter, respectively). However, we stress that PICRUSt, CopyRighter, and PAPRICA aim to correct for the copy number variation when analyzing the composition of 16S rRNA gene Operational Taxonomic Units (OTUs), and were not designed to compute the ACN. Therefore, these tools implement a more complex approach based on the phylogenetic annotation of the 16S rRNA genes.

### Analysis of the average genome size and 16S rRNA gene copy number in TARA oceans metagenomes

**Fig. 4:**
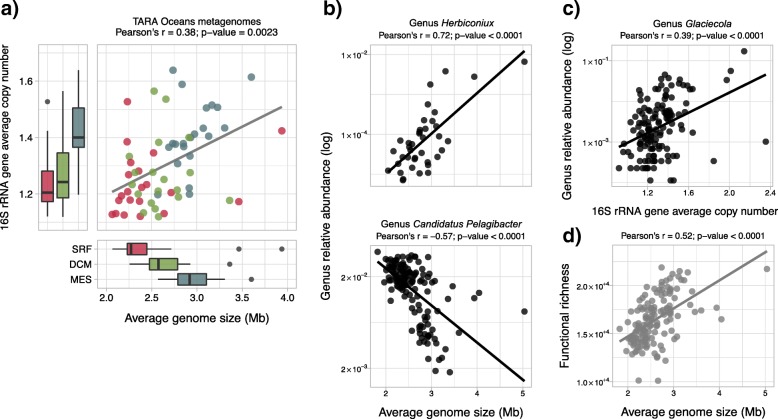
Exploratory analyses performed on TARA Oceans metagenomes. **a** Scatter plot comparing the AGS and ACN in the matching subset of 63 TARA Oceans metagenomes representing the surface, deep chlorophyll maximum and mesopelagic water layers (SRF, DCM, and MES, respectively) in 21 sampling sites. The box plots in the lower and left-hand side panels show the distributions of the Average Genome Size (AGS) and 16S rRNA gene Average Copy Number (ACN) in the SRF, DCM, and MES water layers. For the sake of clarity, two metagenomes with relatively large AGS or ACN values were not included in the plot. These are the TARA_076_DCM_0.22–3 with an AGS = 5,036,010 bp and TARA_064_DCM_0.22–3 with an ACN = 2.4. **b** Scatter plots comparing the AGS with the log relative abundance of the *Herbiconiux* and *Candidatus Pelagibacter* genera (upper and lower panel, respectively) in TARA Oceans metagenomes. *Herbiconiux* and *Candidatus Pelagibacter* genera had the strongest positive and negative Pearson’s correlations with the AGS, respectively. **c** Scatter plot comparing the ACN with the log relative abundance of the *Glaciecola* genus in TARA Oceans metagenomes. This genus showed the strongest positive Pearson’s correlation with the ACN. The abundance of these genera was computed by Sunagawa et al. based on the annotation of 16S rDNA Operational Taxonomic Units (OTUs). **d** Scatter plot comparing the AGS with the functional richness of TARA Oceans metagenomes. The functional richness was computed by Sunagawa et al. based on the abundance estimation of eggNOG orthologous groups

We computed the AGS and ACN in the 139 prokaryotic metagenomes of TARA Oceans (using complete metagenomic samples) to analyze microorganisms’ ecological strategies associated with different marine environmental conditions. Firstly, we conducted pairwise comparisons of the AGS and ACN between water layers. For this task, we used a matching subset of 63 TARA Oceans metagenomes that represent the surface (SRF), deep chlorophyll maximum (DCM) and mesopelagic (MES) water layers in 21 sampling stations across the globe [28]. The results showed that apart from the ACN of the SRF and DCM water layers, all other water layers have significant differences in their trait values (paired Wilcoxon rank-sum test; all *p*-values < 0.05; see Additional file 10). As observed previously [16], we found that the two traits were significantly correlated among themselves (Pearson’s r = 0.38; *p*-value = 0.0023) (see Fig. 4a; Additional file 11).

The distributions of the ACN values resemble the differences in minimum generation times between water layers, computed by Sunagawa et al. 2015 for the same metagenomes. As shown by Vieira-Silva & Rocha (2010) [17], the 16S rRNA gene copy number has a strong correlation with the growth rate of microorganisms.

Additionally, based on the analysis of the 139 TARA Oceans metagenomes, we observed significant correlations between both traits and the sampling depth in the water column (AGS vs. water depth: Pearson’s r = 0.46; ACN vs. water depth: Pearson’s r = 0.28; all *p*-values < 0.001; Additional file 11). Similar findings relating the genome size and 16S rRNA copy number with water depth have been previously described [47, 48]. We also obtained a significant correlation between the ACN and water temperature after controlling for water depth (Pearson’s r = − 0.34 and *p*-value < 0.001; Additional file 11), which was not observed for the AGS. This result could be explained by the fact that psychrophiles are slower growers than expected, given their growth-associated genomic traits [17]. That is, for the same minimal generation times, organisms inhabiting colder marine environments tend to have a greater 16S rRNA gene copy number to compensate for slower enzymatic activity.

Based on the taxonomic annotation of the rDNA Operational Taxonomic Units (OTUs) obtained by Sunagawa et al. 2015 for TARA Oceans metagenomes, we looked for genera associated with the variability of the AGS and ACN traits. This analysis revealed that the genera *Herbiconiux* and *Candidatus Pelagibacter* had the strongest positive and negative correlations with the AGS, respectively, and the genus *Glaciecola* had the strongest positive correlation with the ACN (see Fig. 4b and c; Additional file 11). *Herbiconiux* species tend to have a relatively large genome size, often above 6 Mbp, while *Candidatus Pelagibacter* has a streamlined genome, which is around 1.3 Mbp. On the other hand, sequenced species of *Glaciecola* contain from four to five rRNA gene operons [49]. These are marine microorganisms with extreme values in their genomic traits, and represent different ecological strategies associated with different environments (see below). We note that we did not find any genus with a significant negative correlation with the ACN. That is, the ACN, which tends to be low, appears to vary between metagenomes, mainly when these have a higher abundance of organisms with a high 16S rRNA gene copy number.

Lastly, we compared the AGS of TARA Oceans metagenomes, with the functional richness estimated by Sunagawa et al. 2015 based on the annotation of eggNOG orthologous groups [33]. We observed a highly significant correlation between these two metagenomic traits (Pearson’s r = 0.52 and *p*-value < 0.0001; see Fig. 4d and Additional file 11). This result was expected, given that the number of genes in prokaryotes is known to be linearly related to the genome size [50]. However, the nature of this relationship using the community AGS and functional richness offers new insights into the ecology of marine microbial communities. The AGS explained a moderate amount of the functional richness variation (R^2^ = 0.27). Several other factors can influence the community functional richness and AGS. For example, the functional richness is also highly correlated with the taxonomic richness and water depth (Pearson’s r = 0.85 and 0.65, respectively; all *p*-values < 0.0001; Additional file 11). A plausible explanation, in line with previous work characterizing prokaryotic ecological strategies [9, 51], is that more heterogeneous environmental conditions, which appear to be found in deeper water layers [28, 52], result in a higher functional and taxonomic richness and more complex ecological interactions. In turn, a higher complexity of the ecological interactions and a greater environmental heterogeneity, increase the demand for larger gene repertories, and consequently, larger genomes.

Taken together, these exploratory analyses indicate that surface marine microbial communities are characterized by a small AGS and low ACN. Such trait values denote the presence of efficient, slow growers and specialist organisms (i.e., k-strategist), in agreement with the oligotrophic environmental conditions commonly found in marine surface waters. On the contrary, microbial communities from the DCM and MES water layers exhibited a larger AGS and greater ACN, which indicate that organisms inhabiting deeper layers tend to have a more diverse metabolism and grow faster (i.e., r-strategist). As such, these organisms respond better to environmental changes and can exploit intensively nutrient rich micro-niches [10, 53, 54].

## Conclusions

In this work, we developed the ags.sh and acn.sh tools that accurately and rapidly compute the average genome size and 16S rRNA gene average copy number in unassembled prokaryotic metagenomes. The quantification of these traits provides a powerful approach to characterize microbial ecological strategies. We benchmarked and evaluated the performance of these tools using simulated metagenomic datasets composed of contrasting microbial communities. In these analyses, we showed that the ags.sh tool is up to 11 times faster with comparable or higher accuracy than MicrobeCensus. Reducing the computation time is a valuable improvement given the large data volumes generated by current sequencing technologies. Ags.sh can be readily used to process a comprehensive metagenomic sample for the estimation of the AGS, as exemplarily applied here on TARA Oceans metagenomes. Given that MicrobeCensus is already a highly accurate tool, there was little room for improvement in this sense, and ags.sh only showed a moderate improvement in accuracy regarding the absolute error rates. However, the fact that ags.sh derives the AGS analytically makes it more reliable in comparison to MicrobeCensus. Lastly, our benchmarking analysis of the acn.sh tool showed that it has remarkable accuracy and outperforms the ACN computation approaches based on the copy number predictions of PICRUSt, CopyRighter, and PAPRICA. The fact that acn.sh is exclusively dedicated to the computation of the ACN allows to considerably simplify the analysis workflow.

The exploratory analyses performed on TARA Oceans metagenomes demonstrate the applicability of our tools to compute the AGS and ACN traits on unassembled metagenomic data, and predict the dominant ecological strategies taking place within microbial communities.

We note that the results presented here, show that the AGS and ACN can be derived analytically based on the annotation of single-copy and 16S rRNA genes. Accordingly, future implementations of the ags.sh and acn.sh tools have the potential to improve in speed and accuracy, as gene annotation tools continue to advance. Additionally, in future implementations, it will be of particular interest to include the computation of the AGS and ACN variances [55].

## Availability and requirements

Project name: AGS-and-ACN tools

Project home page: https://github.com/pereiramemo/AGS-and-ACN-tools

Operating system(s): Platform independent.

Programming language: AWK, Bash, and R.

Other requirements: Docker.

License: GNU General Public License v3.0.

Any restrictions to use by non-academics: none.

## Additional files

Additional file 1: Single-copy genes universally present in prokaryotes. Table showing the COG number of the 35 single-copy genes identified by Raes et al. (2007), and the number of sequences included in each COG. (XLSX 42 kb)

Additional file 2: Reference genome datasets. Excel sheets with the accession, taxid, organism name, and FTP URL of the genomes used to generate the simulated metagenomic datasets. (XLSX 105 kb)

Additional file 3: Simulated metagenomic datasets. Table showing main characteristics describing the simulated metagenomic datasets. (XLSX 10 kb)

Additional file 4: Benchmarking the running time of ags.sh against MicrobeCensus. Table showing the mean running time of ags.sh and MicrobeCensus using 4, 8, and 16 threads, for the estimation of the AGS in five TARA Oceans metagenomes subsampled to two million paired-end reads. (XLSX 35 kb)

Additional file 5: Benchmarking the accuracy of ags.sh against MicrobeCensus: figure illustration. Plots of the Pearson’s correlation coefficients (upper panel) and absolute percentage error (APE) value distributions (lower panel) of the AGS computed by ags.sh and MicrobeCensus, with respect to the reference AGS. The comparisons were performed using the simulated metagenomes of different read length of the General, Infant Gut, and Marine datasets. For the sake of clarity, 70 outlier APE values (2.3% of the total data) were not included in the plot. (PDF 60 kb)

Additional file 6: Benchmarking the accuracy of ags.sh against MicrobeCensus: summary statistics. Table showing the Medians, Means and Standard Deviations of the Average Percentage Error (i.e., MdAPE, MAPE, and SDAPE, respectively), and Pearson’s r correlation coefficients obtained in the analysis benchmarking the accuracy of ags.sh against MicrobeCensus. (XLSX 41 kb)

Additional file 7: Evaluating the running time of acn.sh. Table showing the mean running time of acn.sh, and ags.sh plus acn.sh, using 4, 8, and 16 threads, for the estimation of the ACN in five TARA Oceans metagenomes subsampled to two million paired-end reads. (XLSX 35 kb)

Additional file 8: Benchmarking the accuracy of acn.sh against PICRUSt, CopyRighter, and PAPRICA: figure illustration. Plots of the Pearson’s correlation coefficient (upper panel) and the absolute percentage error (APE) value distributions (lower panel) of the ACN computed by acn.sh, PICRUSt, CopyRighter, and PAPRICA, with respect to the reference ACN. As mentioned above, we compared these tools using simulated metagenomes of different read length of the General, Infant Gut, and Marine datasets. For the sake of clarity, 100 outlier APE values (4.2% of the total data) were not included in the plot. (PDF 62 kb)

Additional file 9: Benchmarking the accuracy of acn.sh against PICRUSt, CopyRighter, and PAPRICA: summary statistics. Table showing the Medians, Means and Standard Deviations of the Average Percentage Error (i.e., MdAPE, MAPE, and SDAPE, respectively), and Pearson’s r correlation coefficients obtained in the analysis benchmarking the accuracy of acn.sh against PICRUSt, CopyRighter, and PAPRICA. (XLSX 38 kb)

Additional file 10: Comparing the AGS and ACN of TARA Oceans metagenomes between water layers. Table showing details of the Wilcoxon rank-sum tests, performed to evaluate the differentiation of TARA Oceans metagenomes from different water layers according to their AGS, and ACN. The comparisons were made on a matching subset of 63 metagenomes, obtained from the surface, deep chlorophyll maximum, and mesopelagic water layers (SRF, DCM, and MES, respectively), at 21 sampling sites. (XLSX 44 kb)

Additional file 11: Exploring the variability of the AGS and ACN of TARA Oceans metagenomes. Table with details of the Pearson correlation tests between and among the AGS and ACN, and the functional and taxonomic richness of TARA Ocean metagenomes, and the water depth and temperature of the sampling sites. (XLSX 34 kb)

## Data Availability

ags.sh and acn.sh are free software distributed under the GNU General Public License v3.0. These tools are available at https://github.com/pereiramemo/AGS-and-ACN-tools, where we also provide their source code. In the Additional file 2, we provide the RefSeq (https://www.ncbi.nlm.nih.gov/refseq) assembly accession number of the genome sequences used to simulate the metagenomic datasets. The metagenomic data analyzed here can be downloaded from https://www.ebi.ac.uk/ena (study accession PRJEB1787).

## Abbreviations

ACN: 16S rRNA gene Average Copy Number
AGS: Average Genome Size
APE: Absolute Percentage Error
DCM: Deep Chlorophyll Maximum
MAGs: Metagenome Assembled Genomes
MdAPEs: Median of the Absolute Percentage Errors
MES: Mesopelagic
NGs: Number of Genomes
ORFs: Open Reading Frames
OTUs: Operational Taxonomic Units
SRF: Surface
