# Supplementary material for: Fast and accurate average genome size and 16S rRNA gene average copy number computation in metagenomic data
Source: BMC Bioinformatics. 2019 Sep 5;20:453. doi: 10.1186/s12859-019-3031-y (PMC6727555; doi:10.1186/s12859-019-3031-y)
Supplement: Supplementary file 5 — Benchmarking the accuracy of ags.sh against MicrobeCensus: figure illustration. Plots of the Pearson’s correlation coefficients (upper panel) and absolute percentage error (APE) value distributions (lower panel) of the AGS computed by ags.sh and MicrobeCensus, with respect to the reference AGS. The comparisons were performed using the simulated metagenomes of different read length of the General, Infant Gut, and Marine datasets. For the sake of clarity, 70 outlier APE values (2.3% of the total data) were not included in the plot. (PDF 60 kb) [file 12859_2019_3031_MOESM5_ESM.pdf]

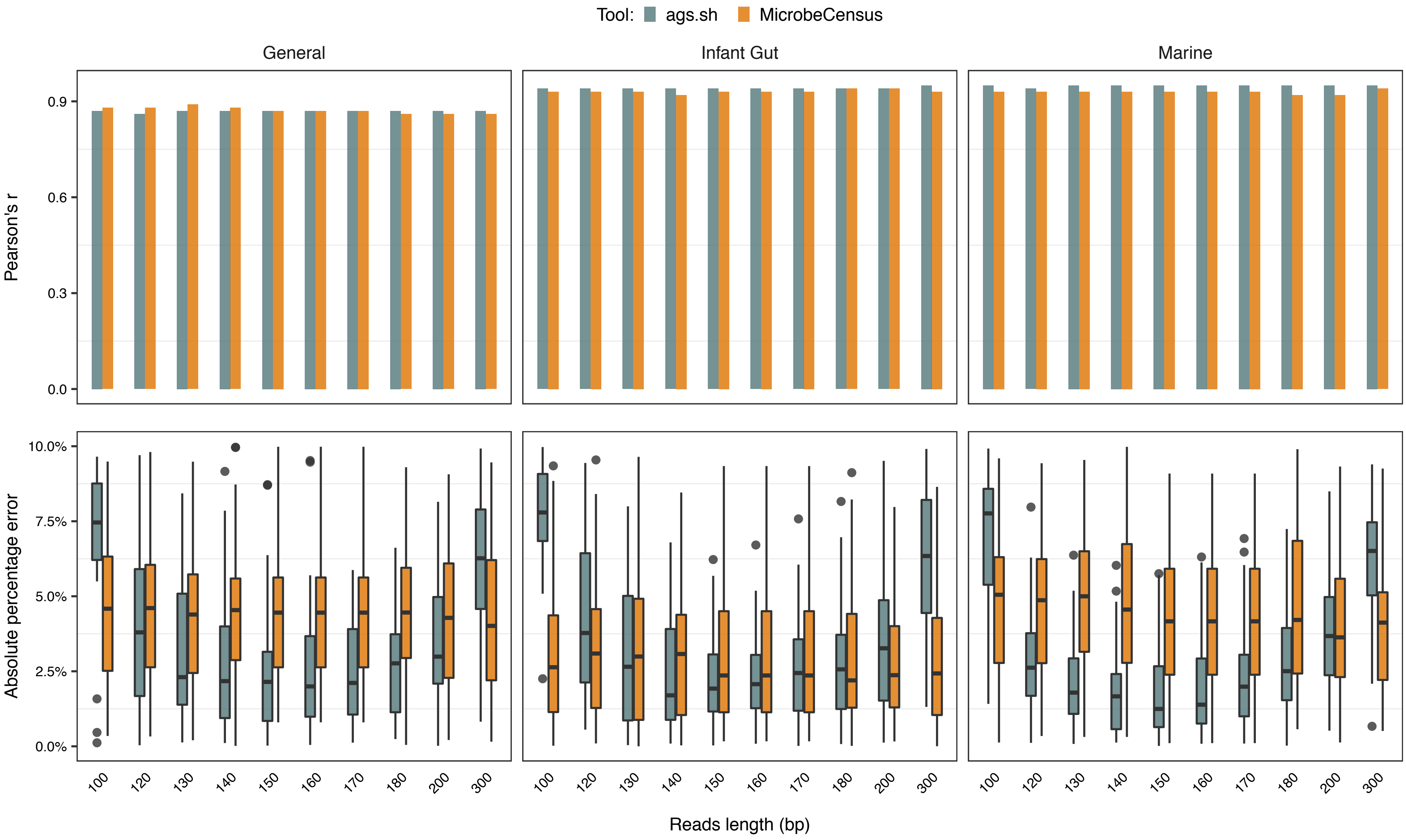

Additional file 5 | Benchmarking the accuracy of ags.sh against MicrobeCensus: figure illustration. Plots of the Pearson's correlation coefficients (upper panel) and absolute percentage error (APE) value distributions (lower panel) of the AGS computed by ags.sh and MicrobeCensus, with respect to the reference AGS. The comparisons were performed using the simulated metagenomes of different read length of the General, Infant Gut, and Marine datasets. For the sake of clarity, 70 outlier APE values (2.3% of the total data) were not included in the plot.
