# Supplementary material for: Fast and accurate average genome size and 16S rRNA gene average copy number computation in metagenomic data
Source: BMC Bioinformatics. 2019 Sep 5;20:453. doi: 10.1186/s12859-019-3031-y (PMC6727555; doi:10.1186/s12859-019-3031-y)
Supplement: Supplementary file 8 — Benchmarking the accuracy of acn.sh against PICRUSt, CopyRighter, and PAPRICA: figure illustration. Plots of the Pearson’s correlation coefficient (upper panel) and the absolute percentage error (APE) value distributions (lower panel) of the ACN computed by acn.sh, PICRUSt, CopyRighter, and PAPRICA, with respect to the reference ACN. As mentioned above, we compared these tools using simulated metagenomes of different read length of the General, Infant Gut, and Marine datasets. For the sake of clarity, 100 outlier APE values (4.2% of the total data) were not included in the plot. (PDF 62 kb) [file 12859_2019_3031_MOESM8_ESM.pdf]

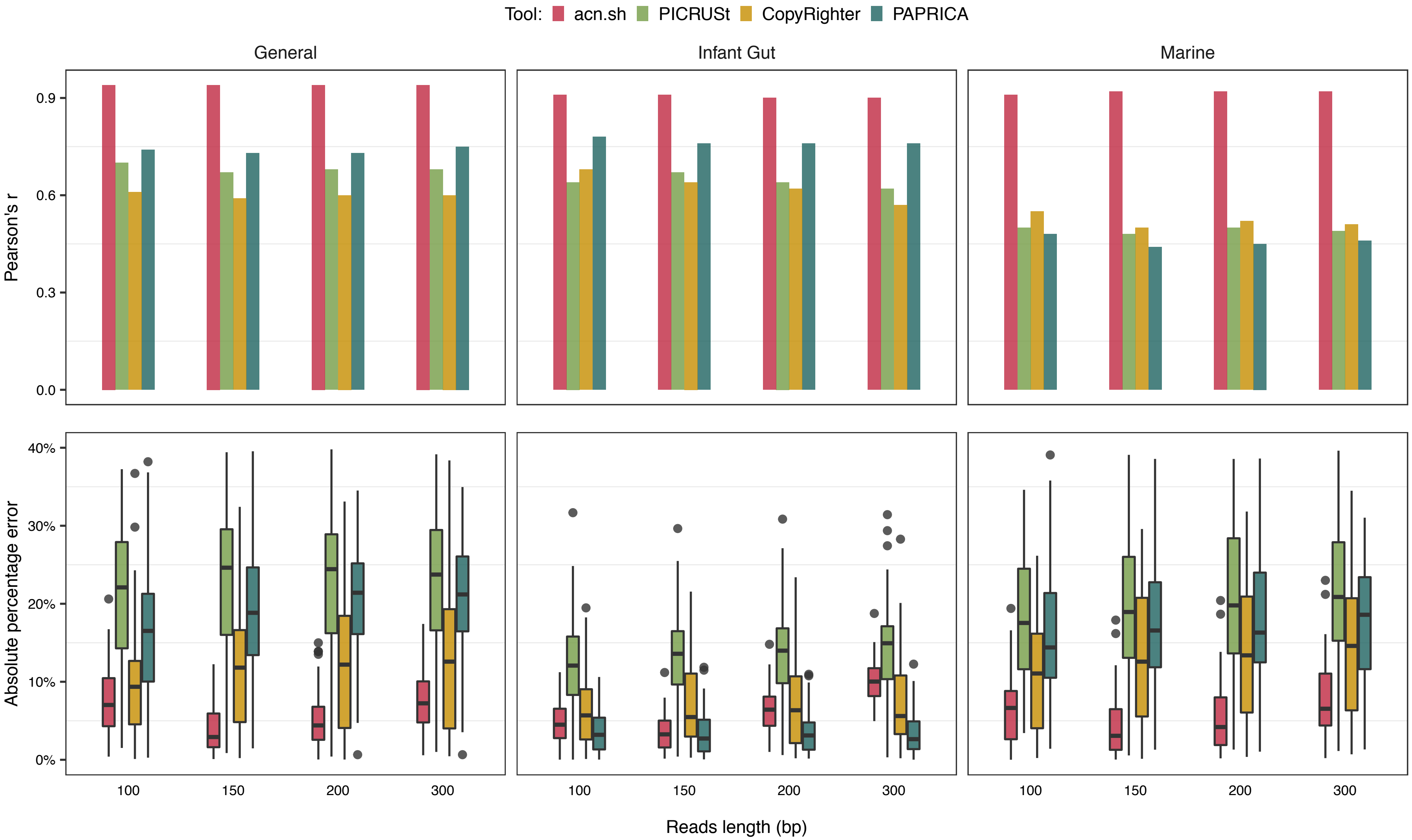

Additional file 8 | Benchmarking the accuracy of acn.sh against PICRUSt, CopyRighter, and PAPRICA: figure illustration. Plots of the Pearson's correlation coefficient (upper panel) and the absolute percentage error (APE) value distributions (lower panel) of the ACN computed by acn.sh, PICRUSt, CopyRighter, and PAPRICA, with respect to the reference ACN. As mentioned above, we compared these tools using simulated metagenomes of different read length of the General, Infant Gut, and Marine datasets. For the sake of clarity, 100 outlier APE values (4.2% of the total data) were not included in the plot.
